# Developing cultural competence in general practitioners: an integrative review of the literature

**DOI:** 10.1186/s12875-016-0560-6

**Published:** 2016-11-15

**Authors:** Kelly Watt, Penny Abbott, Jenny Reath

**Affiliations:** 1 School of Medicine - Campbelltown Campus Building 30, University of Western Sydney, Locked Bag 1797, Penrith, NSW 2751 Australia; 2 School of Medicine - Campbelltown Campus Building 30.3.24, University of Western Sydney, Locked Bag 1797, Penrith, NSW 2751 Australia

**Keywords:** Cultural competency, Cultural competency education, Graduate medical education, General practice

## Abstract

**Background:**

Cultural competence is a broad concept with multiple theoretical underpinnings and conflicting opinions on how it should be materialized. While it is recognized that cultural competence should be an integral part of General Practice, literature in the context of General Practice is limited.

The aim of this article is to provide a comprehensive summary of the current literature with respect to the following: the elements of cultural competency that need to be fostered and developed in GPs and GP registrars; how is cultural competence being developed in General Practice currently; and who facilitates the development of cultural competence in General Practice.

**Methods:**

We conducted an integrative review comprising a systematic literature search followed by a synthesis of the results using a narrative synthesis technique.

**Results:**

Fifty articles were included in the final analysis. Cultural competence was conceptualized as requiring elements of knowledge, awareness/attitudes and skills/behaviours by most articles. The ways in which elements of cultural competence were developed in General Practice appeared to be highly varied and rigorous evaluation was generally lacking, particularly with respect to improvement in patient outcomes. Formal cultural competence training in General Practice appeared to be underdeveloped despite GP registrars generally desiring more training. The development of most aspects of cultural competence relied on informal learning and in-practice exposure but this required proper guidance and facilitation by supervisors and educators. Levels of critical and cultural self-reflection amongst General Practitioners and GP registrars varied and were potentially underdeveloped. Most standalone training workshops were led by trained medical educators however the value of cultural mentors was recognised by patients, educators and GP registrars across many studies.

**Conclusions:**

Cultural competency development of GP registrars should receive more focus, particularly training in non-conscious bias, anti-racism training and critical self-reflectiveness. There is a need for further exploration of how cultural competence training is delivered within the GP training model, including clarifying the supervisor’s role.

It is hoped this discussion will inform future research and training practices in order to achieve quality and respectful care to patients across cultures, and to remove health inequities that exist between cultural groups.

## Background

Health care systems and health care practitioners must recognize and respect the needs of an increasingly diverse population, promoting equity of access and patient safety. Cultural competence is needed to improve the effectiveness of cross-cultural interactions between health services, clinicians and patients [1].

Cultural competence is a broad concept with a variety of views on what constitutes it and how it should be materialized [2]. It is most commonly defined as “a set of consistent behaviours, attitudes and policies that enable a system, agency or individual to work within a cross-cultural context or situation effectively” [1, 3]. Cultural competency curriculum frameworks and models vary considerably in scope, length, content and mode of delivery [4–6]. Furthermore, a wide variety of instruments have been developed to assess cultural competence, each with their own assumptions about what constitutes cultural competence [6, 7]. Despite the strong association between racism and ill health of minority groups, the literature is limited with respect to the prevalence and impact of racism and the effectiveness of approaches to eradicate it [8].

General practice provides “person-centred, continuing, comprehensive and coordinated whole person healthcare to individuals and families in their communities” [9] and therefore cultural competence should be integral [10]. The individual doctor-patient consultation is the main vehicle through which health care is provided in this setting, thus effective cross-cultural interactions are vital. Although there is good evidence to suggest formal training in cultural competence does improve clinicians’ attitudes, knowledge and skills in cultural competence [4, 11], there are few studies conducted in the context of General Practice vocational training.

In Australia General Practice (GP) trainees, or registrars, receive a standard amount of formal teaching, but the majority of their learning is informal and takes place experientially in the workplace under the supervision of accredited GP supervisors [12–14]. Similarly, internationally in the United States (US), United Kingdom (UK) and Europe, GP training generally occurs in a workplace setting where the GP registrar learns while participating in the practice under the supervision of accredited GP supervisors [12]. How cultural competence is developed in GP registrars in this setting is unclear, with much of focus in the literature being on medical student and hospital staff training. The GP supervisor has been described as the most important person involved in the training of Australian General Practitioners, however their role in developing and assessing the cultural competence of GP registrars is unclear [15–17]. A greater understanding of the current cultural competence training literature in the specific context of General Practice will enable better insight into its complexities, further development of effective training models and inform review of current training standards.

The aim of this article is provide a comprehensive summary of the current literature with respect to the following questions:

1. What are the elements of cultural competency that need to be fostered and developed in General Practitioners and GP registrars?
2. How is cultural competence being developed in General Practice currently?
3. Who facilitates the development of cultural competence in General Practice?

## Methods

We conducted an integrative review [18], in order to draw conclusions from studies using diverse research methodologies [18, 19]. The integrative review comprised a systematic literature search of peer- reviewed and grey literature published between 1998 and 2013, followed by narrative synthesis of the results [20, 21]. The publication inclusion dates were chosen because of the substantial change in the structure of GP training programs at that time [22], and because of the marked increase in articles published on the topic from 1998 onwards. The last date searched was April 31st 2013.

A systematic electronic database search strategy was developed in collaboration with a health librarian. The search strategy, including eligibility criteria and information sources, is outlined in Table 1. Our initial search strategy included papers that considered cultural competence and diversity in general practice broadly in order to maximize the articles available for review. Our subsequent review of titles and abstracts refined our search to papers describing cultural competence and its development in General Practice. KW performed the search and selection of articles in collaboration with PA.

**Table 1:** Search strategy

Search Engines:
PUBMED, WEB OF SCIENCE, CINAHL, SCOPIS, ERIC, Google scholar, RACGP website, and a reference list search.
Search Terms (in varying combinations):
Cultural competence (broad and MeSH), cultural safety/awareness, cross-cultural, diversity, multicultural, training, education, post-graduate, primary care, general practice, community, ambulatory, family medicine
Inclusion criteria: English language
• Dated between 1998–2013
• Articles addressing aspects of cultural competence or cultural diversity in primary care or general practice,
o Participants must include any primary care stakeholders, including clinicians, educators, community members and patients
• Empirical research and review or discussion articles
• Peer-reviewed articles and grey literature
Exclusions:
• Articles focusing on specific health interventions
• Articles addressing cultural competence that do not directly relate to primary care or general practice

### Evaluation and analysis

A narrative synthesis was chosen as the most appropriate method of knowledge synthesis as it enables the synthesis of a wide range of different research designs, including both qualitative and quantitative, in a systematic and robust way [19, 20]. It provides at minimum a structured and in depth summary of what is currently known about this topic in order to identify gaps in evidence and recommendations for trainers. It also seeks to further interpret the findings, including explanations and moderators of the findings [20].

We undertook the narrative synthesis according to guidance provided by Popay et al. [20]. We extracted data in narrative form from each article, including study type, participant population, research aims, findings and discussion points. This formed the preliminary synthesis. We then explored relationships in the data, including comparing research findings across multiple studies and developing themes. We finally assessed the robustness of the synthesis as a whole by critically reflecting on the quality and quantity of studies included, minimizing bias and assessing the strength of evidence upon which our conclusions are drawn. [20]. Thematic synthesis of the results and discussion sections of each article was performed to identify important themes across the studies [21]. QSR International’s NVivo 10 software was used to manage the data. Initially, line-by-line coding was performed, followed by the development of descriptive themes. These were then refined and incorporated into the final interpretation.

Each article was critically appraised using criteria for methodological rigor from the “Criteria Appraisal Skills Program (CASP) checklist” [23]. Articles rated as less rigorous were not excluded but their rigor was taken into account in the final analysis. For example, conclusions drawn from higher quality quantitative articles, such as systematic reviews, were prioritized over those from less rigorous evaluative and interventional studies, particularly if the evidence of effectiveness was conflicting. Qualitative articles that were of higher quality contributed more to the overall synthesis because they had more trustworthiness and transferability. With more details on context and more reflection, it allowed us to more confidently assess the importance of their findings. They also tended to contain more highly developed analyses within them. This was particularly important during exploration of relationships, comparing and contrasting data and developing themes across studies [21]. It was necessary to include them due to the broad focus of the review questions, the limited number of papers addressing this issue and the heterogeneity of the studies [20].

The principal researcher KW performed the preliminary selection of articles and synthesis and theme development. The final list of articles for inclusion along with excluded articles of borderline relevance was provided to research team members PA and JR and consensus on the final selection was reached. PA and JR separately reviewed 10 articles each to independently assess eligibility for inclusion in the review and extract themes for comparison with those found by KW. These articles were chosen by KW for independent review by team members based on being of key relevance and having higher complexity of themes. Relationships between study findings and themes were discussed at regular meetings throughout the course of the study amongst the research team members.

## Results

### Article selection

**Fig. 1:**
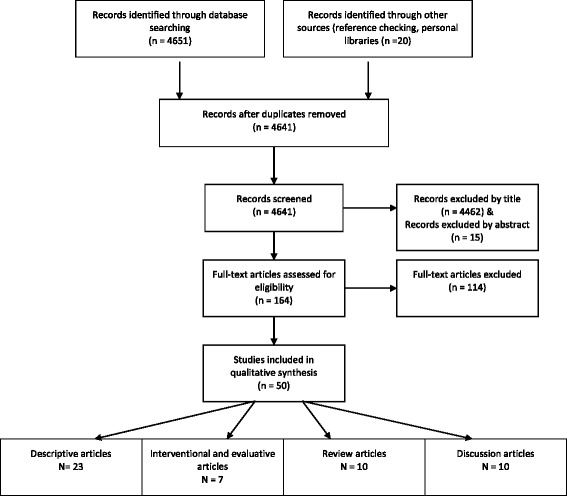
PRISMA flow chart – article selection process

The literature search yielded 4641 titles. Figure 1 displays the article selection process in a PRISMA flow chart. 4462 articles were excluded on the basis of the title and a further 129 articles were excluded based on a review of the abstract (15) and full text (114). Fifty articles met the inclusion criteria and were included in the final analysis after members of the research team reached consensus.

**Fig. 2:**
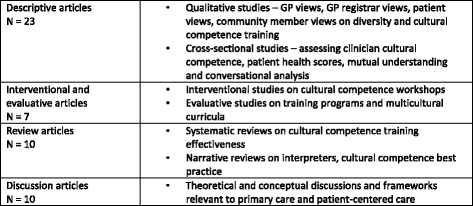
Article Types

The range of article types can be seen in Fig. 2. Qualitative and cross-sectional studies explored views of General Practitioners, GP registrars, patients, culturally diverse health workers, including interpreters and other health providers as well as medical educators and other stakeholders in clinical education such as program directors. Studies used various theoretical frameworks ranging from anthropological, psychological and sociological and behavioural science to linguistics and political science standpoints. Fourteen articles were either based in Australia or included Australian participants [4, 6, 11, 24–34]. Eight of the Australian articles focused on cultural competence specific to Aboriginal and Torres Strait Islander health [24, 27, 29–34]. Twenty-one articles were based in the US [2, 4–6, 11, 35–51] and the remaining articles were from other western countries such as Canada, UK, Sweden, Norway and the Netherlands. Qualitative articles were of good rigor, as were the systematic reviews and theoretical articles. Out of the interventional articles only one was randomized and used outcome measures that were standardized and previously validated [52]. Three studies were from grey literature (one PhD thesis and 2 evaluative reports). All studies were included in the final analysis and the assessments of rigor were taken into account. For example, if articles had conflicting data, both were taken into account, but more weight was given to the article assessed as being of higher rigor.

### Elements of cultural competence

**Fig. 3:**
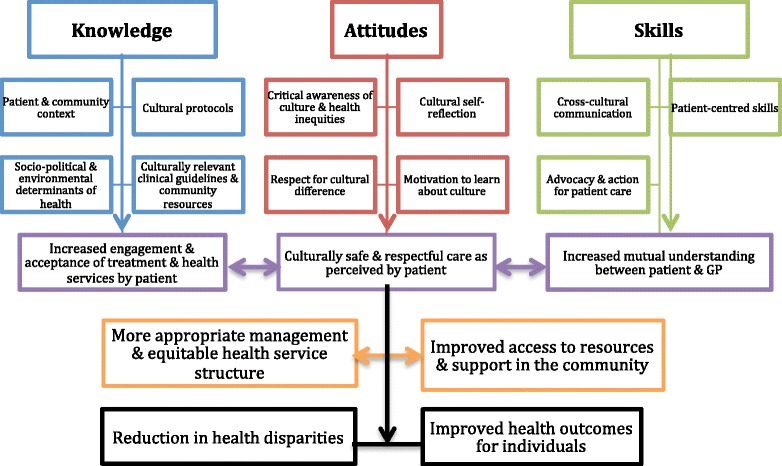
Elements of Cultural Competence

Central to culturally competent practice is the recognition and incorporation of the dynamics of culture effectively into effective health service delivery. Conceptualising individual elements of cultural competence, however, is complex and at times contentious. The majority of articles in this review conceptualized the elements of cultural competence as knowledge, awareness/attitudes and skills/behaviours and highlighted that aspects of all three areas need to be developed in General Practitioners and indeed all primary health care providers. The elements of cultural competence according to the synthesis are summarized in Fig. 3 and are discussed briefly below.

#### Knowledge

In General Practice, knowledge of the local context in which the patient and family is situated is vital, such that developing knowledge should focus on the local community in which the General Practitioner works [24, 29, 32, 41]. Knowledge of a patient’s cultural context was thought to be important by General Practitioners [53–55], GP registrars [30, 36, 56, 57], educators [25, 26], patients [41, 58] and community members [24, 26, 27, 44]. Despite this, General Practitioners often reported accommodating differing cultural values and expectations only when they are explicitly stated by patients [54, 59, 60]. At times they failed to recognise cultural expressions of distress and the effects of immigrant-specific issues on health [61, 62], preferring to focus on individual interpersonal interactions [60, 61].

Stereotyping can occur as a result of cultural training with a narrow focus and using generalisations without awareness of the uniqueness of the individual and the dynamic nature of culture [30, 36, 48, 54]. Medical learners have been reported to focus on differences [57] and to want neat categorical information with high clinical relevance [46]. General Practitioners [54, 61] and GP registrars [30, 36, 56, 57] were found to recognise and fear stereotyping as a potential consequence of cultural competence training, as do medical educators [2, 5, 25, 27, 34, 46–48, 51, 61] In doing so, recognition of cultural difference was often conflated with stereotyping, with limited recognition of the need to test any assumptions held about a patient individually [54, 60, 61].

General Practitioners commonly reported they lacked access to resources such as interpreters and other community health providers, language and culture-appropriate information, and knowledge of access to funding and specific health programs. This was noted to present barriers to culturally competent care and also to training [2, 4, 42, 43]. A lack of knowledge or skills in cross-cultural consultations was also reported to limit motivation to engage in these consultations and to increase stress particularly in GP registrars [36, 56, 61]. GP registrars considered the use of bilingual community health workers to play an important role in mediating cross-cultural interactions, and prioritized this over the need to increase their own consultation skills [57, 63]. Training was associated with increased knowledge and use of appropriate resources [4, 40, 43, 53].

#### Attitudes

Unconscious underlying attitudes and assumptions of General Practitioners can alter their interactions with patients, and in turn the patients’ response to GP care [27, 31, 34, 41, 47, 49, 63]. Addressing underlying racism, assumptions, prejudices and non-conscious bias is therefore a vital component of cultural competence [27, 44, 49, 51, 59, 63] and needs to be directly addressed during training [27, 32, 34, 47].

Critical cultural self-reflection can be seen as the General Practitioner’s ability to recognize the effect of their own position within the power structures of society and within their own culture, and how this affects their interactions [7, 27, 31, 34, 39, 54]. It also involves the ability to adapt in response to this reflection over time, including recognition of deficiencies in practice [25, 53, 54, 57], recognition of their own assumptions, prejudices [27, 36, 54, 56] and non-conscious biases and their effect on the interaction and clinical decision-making [47, 51]. In General Practice, self-reflective professional development may occur without specifically addressing the General Practitioner’s ethno-cultural identity or the influence of this on the consultation [25, 27, 36, 54, 56, 57, 60, 61]. For example General Practitioners may identify cross-cultural consultations as more stressful, but not see how the consultation could be done differently [60]. The concerns some General Practitioners expressed about avoiding stereotyping may result in a failure to address cultural differences and existing biases and assumptions [54, 60, 61, 64].

Motivation of policy makers, medical educators, trainers and GP registrars was found to be a driving factor in cultural competence training and a lack of motivation was shown to be a barrier to its implementation and effectiveness [24, 26, 27, 37, 42, 46]. In environments where non-compulsory training exists, cultural competence training was thought to be under-prioritised or overlooked, but where it is compulsory, resistance by health staff attending formal training was perceived to be very difficult to overcome [25, 27, 46]. The ‘buy in’ was perceived to be critical [46] because training directed at developing awareness of privilege/disadvantage, racism and prejudice was recognised as difficult and risking isolation or disengagement of the audience [30].

#### Skills/behaviours

Developing specific cross-cultural skills and behaviours should enable the General Practitioner to facilitate more effective and respectful health care [5, 44, 49]. Communication was the most frequently cited barrier to effective cross-cultural interactions [26, 44, 53, 55, 56, 58–65] and at times had the potential to result in significant adverse outcomes for patients [25, 26, 45, 56, 61]. In cross-cultural consultations, General Practitioners and patients may have less mutual understanding, which can be associated with poorer adherence [65]. General Practitioners may have more retractive styles of consultation in the cross-cultural context involving patients less in decision-making and checking their understanding less often [64]. Communication skills training alone does not necessarily lead to cultural competence [27, 38] and more training in cross-cultural communication skills was shown to be valued and desired by GP registrars [36, 56, 57].

Language differences pertain to a large part of communication difficulties [25, 28, 36, 37, 44, 53, 54, 56–58, 61–63, 65] and are associated with lower patient satisfaction and mutual understanding [39, 44, 58, 62]. General Practitioners and patients perceived language differences to be mostly overcome by the use of interpreters [54, 58] however interpreter availability was sometimes a barrier [37, 63] as was a lack of knowledge of how to access them [2, 4, 42, 43]. Using professionally trained interpreters was shown to reduce the risk of errors and improve outcomes, particularly in mental health scenarios [45]. Despite this, use of untrained informal interpreters such as family members in General Practice remains common [66]. General Practitioners can be apprehensive about using interpreters, particularly with regard to the accuracy of the interpretation and about losing connection and rapport in both nonverbal cues and personal interaction [59, 66]. In one study, GP registrars did not see value in specific training in the use of interpreters and instead preferred to rely on other health workers being available to assist [57]. Limited time and resources were frequently a barrier to cultural competence, especially as cross-cultural consultations were perceived to be more complex and require much longer consultations [24, 36, 37, 42, 53, 59, 63].

General Practitioners must also be trained to recognise and adapt to different cultural expressions of distress [61], cultural protocols [32, 36, 49, 50, 56, 57, 59] that act as demonstrations of respect, and nonverbal cues and behaviours [25, 36, 41, 53, 55, 59, 61], including those produced by non-conscious biases in the General Practitioner [47]. Many recognised patient-centred skills such as the ability to negotiate, build trust and rapport with patients, eliciting patient models of illness are all patient-centred techniques that are valuable in culturally competent practice [46, 48–51]. Being able to explore culture within the consultation in a respectful and effective way is something General Practitioners often avoid for fear of stereotyping [54, 57, 60, 61] or find difficult [25, 36, 37, 53, 56, 57, 59, 63].

A lack of cultural competence of health services and systems is thought to impede the ability of an individual to provide culturally competent care [27, 32, 34, 44, 54, 55, 63]. Proactively working to effect positive change not only in one’s personal practice but in the wider health and societal systems was thought to be an important aspect of cultural competence [27, 32, 34, 44, 54, 55, 63].

### How is cultural competency developed?

The ways in which the elements of cultural competence are developed in General Practice are highly varied and rigorous evaluation is generally lacking [2, 4, 11, 43].

Formal cultural competence training in General Practice appears to be underdeveloped and inconsistent, and the development of most aspects of cultural competence relies on informal learning, experiential and in-practice exposure [30, 36, 37, 40, 42, 56, 57]. Generally there is a lack of focus on cross-cultural issues within GP training with suggestions that time pressures, political will and competing priorities tend to restrict what GP registrars can learn [26, 27, 36, 37, 42, 56, 57]. GP registrars were found to generally desire more training and often viewed cross-cultural consultations as more stressful due to their perceived lack of confidence, knowledge and skills in this area [37, 56, 57, 63].

Exposure to cultural diversity during training and experience over time has been shown to be valuable in the development of cultural competency [30, 38, 40, 42, 44, 67] and are particularly relevant in the GP training setting. Development of cultural competence of both the individual and the practice or system can be a synergistic process [40] and exposure to diversity can act as a trigger and motivation for learning [36, 57]. Exposure to diversity alone without appropriate facilitation of learning and skills development, may lead to GP registrars developing ad hoc coping behaviours rather than cultural competence [7, 36]. There is also the risk of perpetuating existing barriers to patient care through modeling culturally incompetent attitudes, knowledge and skills of other staff or clinical supervisors [27, 57]. The amount of formal training appears to increase GP registrars’ preparedness and competence to provide cross-cultural care more so than having good role models or exposure to greater diversity cross-cultural case mix throughout training [35].

Integrating cultural competency with the wider curriculum is preferred by both GP registrars [30, 36, 56, 57] and medical educators [5, 32, 46, 50]. This, along with a longitudinal process of development, consolidates and gives more clinical relevance to learning, emphasises the necessity of developing cultural competency for good patient care, and reinforces cultural competency learning as a lifelong process [5, 46, 50, 56].

Interventional studies evaluating formal training in the form of standalone workshops have shown such training improves awareness, knowledge and skills of participants [4, 6, 11, 25, 43, 52, 67], and that this can improve patient satisfaction [11] and mutual understanding [67]. However there is limited research to suggest it improves patient health outcomes [4, 6, 11, 31, 43]. Formal workshops have the additional benefits of high participant satisfaction, efficacy for cost and time, and they can provide standardization of training amongst GP registrars, in a field where training environments can be very different [29, 30, 32]. There were no particular features of formal training programs (such as length of training, content and curriculum and types of training methods used) that were associated with better outcomes [4, 6, 11, 43]. It was recognized however that standalone workshops and the improvements in individual participant knowledge, awareness and skills would not necessarily result in long-term improvements in patient health without wider systemic and organizational changes and support [27, 32, 34, 64].

Types of training suggested by GP registrars included videotaped consultations, role plays and case discussions, community-oriented project work and lectures and training led by representatives from those cultures [36, 56, 57]. GP registrars also tended to desire more exposure to cross-cultural practice, more interpreters, and increasing diversity and awareness of faculty [36, 56, 57]. However GP registrars in one study were ambivalent about formal training because of a fear of that training resulting in increased stereotyping [36]. Cultural mentors and community members preferred informal settings and small group learning during training, as well as narrative and community site visits and cultural immersion [24].

The complexities of conceptualizing cultural competence make assessment of attitudes, knowledge and skills difficult [2, 7]. Instruments and measures of what constitutes cultural competence often make assumptions or reflect biases [7, 41, 46]. However, assessment of cultural competency amongst GP registrars can drive motivation to learn and can be reflective of a supportive educational environment [37, 57]. GP registrars tended to interpret the lack of assessment in this area to mean it was a low priority for learning [36, 37].

The importance of training evaluation cannot be understated, both in terms of ensuring quality and improvement of teaching practices and adding to evidence base [2, 4, 5, 46]. It has been noted that studies evaluating formal training interventions lack methodological rigour, do not adequately control for potential confounding variables (societal factors, external barriers), are difficult to generalize to other settings and tend not to assess patient perspectives or outcomes [4, 6, 11, 31, 43]. Most evaluation studies of educational interventions were process-oriented, although evaluation of complex behaviours must be multi-faceted [5]. Multiple confounders are often present and must be taken into account, such as other social and environmental determinants of health, access and other systemic barriers beyond the control of individual clinical interactions, and this makes evaluation even more difficult [4]. A proposed algorithm for evaluation of educational interventions on patient outcomes exists to incorporate this understanding [4].

### Who facilitates the development of cultural competency?

The majority of interventional studies assessed standalone workshops led by trained medical educators [6, 11, 25, 28, 52, 67]. Experience and training specifically in cultural competence education was perceived to be required by educators given the complexity of the subject [29, 32, 33, 42, 52].

Cultural competency training is thought to risk perpetuating those myths, biases and stereotypes that exist within society already [34, 57], particularly if it is done without the direct input and guidance by the cultural group under consideration. Therefore the value of cultural mentors is recognised by patients, educators and GP registrars across many studies [27, 29, 30, 32, 33, 36, 40, 44, 57]. Cultural mentors are recognised as representatives from their community, able to share their expertise while facilitating partnerships with communities and health care providers [29, 32, 57]. Ensuring cultural mentors and other community members play a central role in training also respects community ownership of cultural knowledge [29, 34]. Currently GP registrar access to cultural mentoring is limited and needs to be expanded [30, 33].

Barriers to the involvement of community members in GP registrar training include having to manage negative attitudes of learners, conflicting family and community commitments, and lack of confidence and experience in training. There is however a desire within culturally diverse communities to engage in training with GP registrars and this should be supported [24, 27, 29, 33]. Having both a medical educator and community member present may improve engagement of GP registrars with the training session, by promoting its relevance and significance to clinical practice [29].

Staff diversity (including interpreters and allied health providers) within individual training practices has been shown to provide GP registrars with opportunities for cultural education [36, 40, 44, 53]. However, at times this may create a reliance on diverse staff to provide care for those patients, allowing other staff to avoid developing their own cultural competence [27, 57].

GP supervisors often functioned as role models [25] and access to good role models was associated with greater preparedness to provide cross-cultural care [35] although a lack of access to such role models was noted [36, 37, 57]. Supervisors reported that addressing cultural and communication issues was often difficult because of the risk of appearing to be racist [25]. They often felt ill equipped to address these issues [25]. Only one article was found specifically addressing GP supervisors and cultural competency, reflecting a paucity of evidence in this area.

## Discussion

This is the first integrative review to synthesize current themes and evidence on cultural competence and cultural competence training specifically in the context of General Practice.

Cultural competence is complex and multifaceted, requiring the General Practitioner to have a combination of equally important knowledge, attitudes and skills, in order to produce a safe, respectful experience for the patient and an effective consultation resulting in better health outcomes [68]. Studies exploring General Practitioners and GP registrar views and experiences of cross-cultural practice revealed that training in cultural competence was generally lacking, but desired and deemed important by GP registrars. This suggests there is general willingness for GP registrars to undertake further training, but they require more resources and support and facilitation of this training by clinical role models, medical educators and culturally diverse staff and community members. The central role of the GP supervisor in GP registrar training (through role modelling, mentoring and clinical supervision) suggests that GP supervisors do have a role in developing the cultural competence of their GP registrars, but that role needs to be further elucidated and developed [15–17].

The educational environment can be either a facilitator or barrier to this training. This may be reflected in the motivation and skills of educational staff, as well as the use of assessment as a driver of learning. There appeared to be varying levels of critical self-reflection amongst General Practitioners and GP registrars in general [25, 27, 36, 54, 56, 57, 60, 61]. Training in non-conscious bias, anti-racism training and cultural self-reflectiveness should therefore receive more focus. Consideration must be given to the complexities of teaching these particularly sensitive topics, as well as the potential to alienate audiences or inadvertently perpetuate stereotypes during training. Involvement of cultural mentors and experienced educators trained in this area may reduce these risks [27, 32, 34].

The literature suggests that much of the development of cultural competence in General Practice occurs informally, as fits with the work-place based training common to most GP programs. However, best practice delivery of cultural competence training in this setting has not been well explored. As many studies suggest, cultural competence training is a lifelong process and formal training, such as workshops, is only an introduction [5, 27, 46, 50, 68–70]. Development of cultural competence in GP registrars requires use of a range of strategies, integrated within the curriculum and facilitated by cultural mentors and medical educators experienced in this area [71].

Many of the training approaches described in the literature are likely to be useful in General Practice vocational training. For example, community visits and cultural immersion may correspond to nursing home and home visit consultations during training. Case discussions and role plays form part of the current training approaches led by GP supervisors, however discussion regarding culture and its impact on the consultation tends to be ad hoc and confined within a framework of the patient-centred model [15, 16]. How training should and does proceed from there, how integration should occur into the general curriculum, and where the focus should lie at different stages of training has not been explored. An exploration of racism and its prevalence and impacts on patient care within General Practice will also help in understanding what other influences exist on GP registrar training in this area [8, 72].

There was a lack of empirical evidence on impact of cultural competence on patient outcomes in papers included in this review. In fact, few articles focused on patient perspective [26, 41]. This is particularly concerning given the importance of the patient’s perspective in defining a “culturally safe” clinical service [1, 70]. Assessment of the clinicians’ cultural competence from the patient’s perspective should be included, since one of the main goals of cultural competence training is provision of culturally safe health care. Given the lack of methodological rigor in most interventional studies, training interventions should be rigorously evaluated according to the proposed standardized evaluation algorithms in the literature [4].

Further exploration is required of the integration of patient-centred and culturally competent approaches in General Practice. The patient-centred model is well developed in general practice, and although there are many overlapping, possibly synergistic learning skills, between the two paradigms, the focus is ultimately different [51]. Further exploration is required to determine whether a patient-centered approach incorporating elements of cultural competency provides a culturally safe experience for the patient and assist in reducing health inequalities [43]. We further recommend that cultural competency development of GP registrars should receive more focus, particularly training in non-conscious bias, anti-racism training and critical self-reflectiveness; the GP supervisors’ role in developing cultural competency of GP registrars should be further elucidated and developed; and finally, further research should explore how cultural competency can be best developed within the GP training model, and where the focus should lie at different stages of training.

### Limitations

The element of subjectivity during the synthesis process is a limitation, despite measures such as independent evaluation of articles, and constant clarification and discussion of themes with the research team.

Given the authors are based in Australia, a greater knowledge of Australian-based grey literature may have meant some international grey literature was missed. It is also difficult to confirm generalizability amongst the different studies as many were undertaken in different countries and involved differing GP training models. However, the workplace-based training model is common across the vast majority postgraduate medical training programs, where informal learning makes up a large component of General Practice training throughout rotations in both hospital specialties and in GP training practices [12, 14]. Multiculturalism and increasing patient diversity is a common development in General Practice internationally [65, 73].

Including all studies despite varying standards of rigour, including grey literature, was necessary in order to allow for a more comprehensive presentation on a topic with considerable heterogeneity in the literature but limited standardized evidence-based evaluations (61). Assessments of rigour were taken into account as part of the preliminary synthesis, which is one of the strengths of narrative synthesis [19].

## Conclusion

Cross-cultural consultations can be stressful and complex for GP registrars and General Practitioners alike. Formal cultural competence training in General Practice is generally lacking, despite the recognition that it is of vital importance and that GP registrars generally desire this. There is a need for further exploration of how cultural competence training is delivered via the informal curriculum, and whether this is effective. Increased training focus on non-conscious bias, anti-racism training and self-reflectiveness is required.

The ultimate end point of developing cultural competence in any clinician should be not only to provide quality and respectful care to patients across cultures, but also to reduce racism, discrimination and remove the health inequities that exist between cultural groups.

It is hoped that the discussion of this broad and complex topic will better inform future training practices including curriculum development, and implementation for GP registrars.

## Abbreviations

GP: General Practice
UK: United Kingdom
US: United States

## References

[1] National Health and Medical Research Council (2006). Cultural competency in health: a guide for policy, partnerships and participation.

[2] Chun MB, Takanishi DM (2009). The need for a standardized evaluation method to assess efficacy of cultural competence initiatives in medical education and residency programs. Hawaii Med J.

[3] Cross TL, Bazron BJ, Dennis KW, Isaacs MR. Towards a Culturally Competent System of Care: A Monograph on Effective Services for Minority Children Who Are Severely Emotionally Disturbed, vol. 1. Washington: Child and Adolescent Service System Program (CASSP) Technical Assistance Center; 1989.

[4] Lie DA, Lee-Rey E, Gomez A, Bereknyei S, Braddock CH (2011). Does cultural competency training of health professionals improve patient outcomes? A systematic review and proposed algorithm for future research. J Gen Intern Med.

[5] Betancourt JR (2003). Cross-cultural medical education: conceptual approaches and frameworks for evaluation. Acad Med.

[6] Chipps JA, Simpson B, Brysiewicz P (2008). The effectiveness of cultural-competence training for health professionals in community-based rehabilitation: a systematic review of literature. Worldviews Evid Based Nurs.

[7] Kumas-Tan Z, Beagan B, Loppie C, MacLeod A, Frank B (2007). Measures of cultural competence: examining hidden assumptions. Acad Med.

[8] Paradies Y, Harris RIA (2008). The Impact of racism on indigenous health in Australiand Aotearoa: towards a research agenda. Discussion Paper No 4.

[9] Definition of general practice [http://www.racgp.org.au/whatisgeneralpractice.]. Accessed 16 Mar 2014.

[10] Royal Australian College of General Practitioners (2011). Royal Australian college of general practitioners curriculum for Australian general practice 2011. Multicultural Health.

[11] Beach MC, Price EG, Gary TL, Robinson KA, Gozu A, Palacio A, Smarth C, Jenckes MW, Feuerstein C, Bass EB (2005). Cultural competence. Med Care.

[12] Wearne S, Dornan T, Teunissen P, Skinner T (2012). General practitioners as supervisors in postgraduate clinical education: an integrative review. Med Educ.

[13] Hays RB, Morgan S (2011). Australian and overseas models of general practice training. Med J Aust.

[14] Roberts RG, Hunt VR, Kulie TI, Schmidt W, Schirmer JM, Villanueva T, Ruth Wilson C (2011). Family medicine training—the international experience. Med J Aust.

[15] Watt K, Abbott P, Reath J (2015). Cultural competency training of GP Registrars-exploring the views of GP Supervisors. Int J Equity Health.

[16] Watt K, Abbott P, Reath J. Cross-cultural training of general practitioner registrars: how does it happen? Aust J Prim Health. 2015;22(4):349–53. 10.1071/PY14165 28442029

[17] Abbott P, Reath J, Gordon E, Dave D, Harnden C, Hu W, Kozianski E, Carriage C (2014). General practitioner supervisor assessment and teaching of registrars consulting with aboriginal patients-is cultural competence adequately considered?. BMC Med Educ.

[18] Whittemore R, Knafl K (2005). The integrative review: updated methodology. J Adv Nurs.

[19] Lucas PJ, Baird J, Arai L, Law C, Roberts HM (2007). Worked examples of alternative methods for the synthesis of qualitative and quantitative research in systematic reviews. BMC Med Res Methodol.

[20] Popay J, Roberts H, Sowden A, Petticrew M, Arai L, Rodgers M, Britten N, Roen K, Duffy S. Guidance on the conduct of narrative synthesis in systematic reviews. A product from the ESRC methods programme Version 1. 2006.

[21] Thomas J, Harden A (2008). Methods for the thematic synthesis of qualitative research in systematic reviews. BMC Med Res Methodol.

[22] Australian General Practice - A celebration [http://www.racgp.org.au/yourracgp/organisation/history/college-history/australian-general-practice/]. Accessed 25 Mar 2016.

[23] Critical Appraisal Skills Program (CASP) (2014). CASP checklist.

[24] Nguyen HT, Gardiner A (2008). Indigenous community members as teachers of indigenous health. Aust Fam Physician.

[25] Anderson-Wurf J (2012). A critical appraisal of the cross-cultural supervision of international medical graduate registrars in general practice.

[26] Johnstone MJ, Kanitsaki O (2007). Health care provider and consumer understandings of cultural safety and cultural competency in health care: an Australian study. J Cult Divers.

[27] Fredericks BL (2008). The need to extend beyond the knowledge gained in cross-cultural awareness training. Aust J Indigenous Educ.

[28] Duncan GF, Gilbey D (2007). Cultural and communication awareness for general practice registrars who are international medical graduates: a project of CoastCityCountry Training. Aust J Rural Health.

[29] O’Shea A, Royal Australian College of General Practitioners (2010). Cultural safety training: identification of cultural safety training needs. Cultural safety training.

[30] URBIS (2008). Evaluation of GPET’s aboriginal and Torres strait islander health training framework: final report.

[31] Downing R, Kowal E, Paradies Y (2011). Indigenous cultural training for health workers in Australia. Int J Qual Health Care.

[32] Farrelly T, Lumby B (2009). A best practice approach to cultural competence training. Aboriginal ISL Health Work J.

[33] Martin ME, Reath JS (2011). General practice training in aboriginal and Torres Strait islander health. Med J Aust.

[34] Downing R, Kowal E (2011). A postcolonial analysis of indigenous cultural awareness training for health workers. Health Sociol Rev.

[35] Greer JA, Park ER, Green AR, Betancourt JR, Weissman JS (2007). Primary care resident perceived preparedness to deliver cross-cultural care: an examination of training and specialty differences. J Gen Intern Med.

[36] Park ER, Betancourt JR, Kim MK, Maina AW, Blumenthal D, Weissman JS (2005). Mixed messages: residents’ experiences learning cross-cultural care. Acad Med.

[37] Weissman JS, Betancourt J, Campbell EG, Park ER, Kim M, Clarridge B, Blumenthal D, Lee KC, Maina AW (2005). Resident physicians’ preparedness to provide cross-cultural care. JAMA.

[38] Saha S, Perrin N, Gerrity M, Gatchell M. Measuring physician cultural competence: results from a national survey. J Gen Intern Med. 2010;25:329–9.

[39] Paez KA, Allen JK, Beach MC, Carson KA, Cooper LA (2009). Physician cultural competence and patient ratings of the patient-physician relationship. J Gen Intern Med.

[40] Paez KA, Allen JK, Carson KA, Cooper LA (2008). Provider and clinic cultural competence in a primary care setting. Soc Sci Med.

[41] Tucker CM, Mirsu-Paun A, Van den Berg JJ, Ferdinand L, Jones JD, Curry RW, Rooks LG, Walter TJ, Beato C (2007). Assessments for measuring patient-centered cultural sensitivity in community-based primary care clinics. J Natl Med Assoc.

[42] Culhane-Pera KA, Like RC, Lebensohn-Chialvo P, Loewe R (2000). Multicultural curricula in family practice residencies. Fam Med.

[43] Renzaho AM, Romios P, Crock C, Sonderlund AL (2013). The effectiveness of cultural competence programs in ethnic minority patient-centered health care--a systematic review of the literature. Int J Qual Health Care.

[44] Brach C, Fraser I (2000). Can cultural competency reduce racial and ethnic health disparities? a review and conceptual model. Med Care Res Rev.

[45] Flores G (2005). The impact of medical interpreter services on the quality of health care: a systematic review. Med Care Res Rev.

[46] Betancourt JR, Green AR (2010). Commentary: linking cultural competence training to improved health outcomes: perspectives from the field. Acad Med.

[47] Stone J, Moskowitz GB (2011). Non-conscious bias in medical decision making: what can be done to reduce it?. Med Educ.

[48] Kleinman A, Benson P (2006). Anthropology in the clinic: the problem of cultural competency and how to fix it. PLoS Med.

[49] Pratt HD, Apple RW (2007). Cross-cultural assessment and management in primary care. Prim Care.

[50] Carrillo JE, Green AR, Betancourt JR (1999). Cross-cultural primary care: a patient-based approach. Ann Intern Med.

[51] Saha S, Beach MC, Cooper LA (2008). Patient centeredness, cultural competence and healthcare quality. J Natl Med Assoc.

[52] Harmsen H, Bernsen R, Meeuwesen L, Thomas S, Dorrenboom G, Pinto D, Bruijnzeels M (2005). The effect of educational intervention on intercultural communication: results of a randomised controlled trial. Br J Gen Pract.

[53] Papic O, Malak Z, Rosenberg E (2012). Survey of family physicians’ perspectives on management of immigrant patients: attitudes, barriers, strategies, and training needs. Patient Educ Couns.

[54] Beagan BL, Kumas-Tan Z (2009). Approaches to diversity in family medicine “I have always tried to be colour blind”. Can Fam Physician.

[55] Diaz E, Hjorleifsson S (2011). Immigrant general practitioners in Norway: a special resource? A qualitative study. Scand J Public Health.

[56] Pieper HO, MacFarlane A (2011). “I’m worried about what I missed”: GP registrars’ views on learning needs to deliver effective healthcare to ethnically and culturally diverse patient populations. Educ Health.

[57] Kai J, Bridgewater R, Spencer J (2001). “Just think of TB and Asians”, that’s all I ever hear’: medical learners’ views about training to work in an ethnically diverse society. Med Educ.

[58] Wiking E, Saleh-Stattin N, Johansson SE, Sundquist J (2009). Immigrant patients’ experiences and reflections pertaining to the consultation: a study on patients from Chile, Iran and Turkey in primary health care in Stockholm, Sweden. Scand J Caring Sci.

[59] Fatahi N, Hellstrom M, Skott C, Mattsson B (2008). General practitioners’ views on consultations with interpreters: a triad situation with complex issues. Scand J Prim Health Care.

[60] Wachtler C, Brorsson A, Troein M (2006). Meeting and treating cultural difference in primary care: a qualitative interview study. Fam Pract.

[61] Rosenberg E, Richard C, Lussier MT, Abdool SN (2006). Intercultural communication competence in family medicine: lessons from the field. Patient Educ Couns.

[62] Harmsen JA, Bernsen R, Bruijnzeels M, Meeuwesen L (2008). Patients’ evaluation of quality of care in general practice: what are the cultural and linguistic barriers?. Patient Educ Couns.

[63] Begg H, Gill PS (2005). Views of general practitioners towards refugees and asylum seekers: an interview study. Divers Health Soc Care.

[64] Schouten BC, Meeuwesen L, Harmsen HA (2009). GPs’ interactional styles in consultations with Dutch and ethnic minority patients. J Immigr Minor Health.

[65] Harmsen H, Meeuwesen L, van Wieringen J, Bernsen R, Bruijnzeels M (2003). When cultures meet in general practice: intercultural differences between GPs and parents of child patients. Patient Educ Couns.

[66] Gray B, Hilder J, Stubbe M (2012). How to use interpreters in general practice: the development of a New Zealand toolkit. J Prim Health Care.

[67] Chudley S, Skelton J, Wall D, Jones E (2007). Teaching cross-cultural consultation skills: a course for UK and internationally trained general practice registrars. Educ Prim Care.

[68] Seeleman C, Suurmond J, Stronks K (2009). Cultural competence: a conceptual framework for teaching and learning. Med Educ.

[69] Chun MBJ (2010). Pitfalls to avoid when introducing a cultural competency training initiative. Med Educ.

[70] Kumagai AK, Lypson ML (2009). Beyond cultural competence: critical consciousness, social justice, and multicultural education. Acad Med.

[71] Reath J, Abbott P, Kurti L, Morgan R, Martin M, Parry A, Anning B, Gordon E, Brooker R (2013). Building aboriginal and Torres Strait islander cultural education and cultural mentoring capacity.

[72] Ewen S, Mazel O, Knoche D (2012). Exposing the hidden curriculum influencing medical education on the health of Indigenous people in Australia and New Zealand: the role of the Critical Reflection Tool. Acad Med.

[73] Dogra N, Reitmanova S, Carter-Pokras O (2010). Teaching cultural diversity: current status in U.K., U.S., and Canadian medical schools. J Gen Intern Med.

